# Comparative Evaluation of the Effect of Different Beverages on Color Stability of Two Single-Shade Composite Resins: An In Vitro Study

**DOI:** 10.7759/cureus.109012

**Published:** 2026-05-17

**Authors:** Rachana Hiremath, Rakshith Guru, Santhosh Sathyanarayan, Dwarakananda Bukya, Lokesh Kanchan, Tushar

**Affiliations:** 1 Prosthodontics, Crown and Bridge, Employees’ State Insurance Corporation (ESIC) Dental College, Kalaburagi, IND

**Keywords:** beverages, colour stability, omnichroma, single-shade composite, vittra aps uniqe

## Abstract

Background

Color stability is a critical property for the long-term success of aesthetic composite restorations. Recently introduced single-shade composite resins, such as Omnichroma (Tokuyama Dental, Tokyo, Japan) and Vittra APS Unique (FGM Dental Group, Joinville, Santa Catarina, Brazil), exhibit a chameleon effect that enhances shade matching. However, their resistance to staining from commonly consumed beverages remains inadequately explored.

Aim

The aim of this in vitro study was to comparatively evaluate the effect of different commonly consumed beverages on the color stability of two single-shade composite resins: Omnichroma and Vittra APS Unique.

Materials and methods

This in vitro study included 96 composite disc specimens (n=48 per material), fabricated with standardized dimensions (10 mm diameter and 2 mm thickness). Specimens of each material were divided into four subgroups (n=12) based on immersion media: artificial saliva, turmeric solution, tea, and soft drinks. Baseline color measurements were recorded using a spectrophotometer based on Commission Internationale de l'Eclairage (CIELAB) parameters (L*, a*, b*). Samples were immersed in the respective beverages for 28 days, simulating long-term clinical exposure. Post-immersion color measurements were obtained, and color change (ΔE) was calculated. Statistical analysis was performed using the Shapiro-Wilk test, Wilcoxon signed-rank test, and Mann-Whitney U test, with significance set at p<0.05.

Results

All specimens exhibited statistically significant color changes after immersion in the tested beverages (p<0.05). Among the tested solutions, turmeric demonstrated the highest staining potential, followed by tea, while soft drinks exhibited the least discoloration. In artificial saliva, Omnichroma showed a significantly higher color change (ΔE=17.37±2.79) compared to Vittra APS Unique (ΔE=10.58±0.11) (p<0.001). In contrast, turmeric caused significantly greater discoloration in Vittra APS Unique (ΔE=30.00±4.69) than in Omnichroma (ΔE=18.60±2.47) (p<0.001). Similarly, tea resulted in higher color change in Vittra APS Unique (ΔE=17.80±4.38) compared to Omnichroma (ΔE=5.98±1.39), indicating increased susceptibility to staining. Soft drinks caused relatively minimal discoloration, with no statistically significant difference between the two materials (p>0.05). Overall, Vittra APS Unique demonstrated greater susceptibility to staining in highly pigmented solutions, whereas Omnichroma exhibited comparatively better color stability under similar conditions.

Conclusion

Within the limitations of this in vitro study, both single-shade composite resins were affected by exposure to staining beverages. Turmeric showed the greatest staining effect, followed by tea, while soft drinks had minimal impact on color stability. Omnichroma demonstrated superior resistance to discoloration compared to Vittra APS Unique in highly staining environments. These findings have important clinical implications in guiding the selection of restorative materials, particularly in patients with dietary habits that predispose restorations to staining.

## Introduction

The growing emphasis on aesthetic dentistry has significantly increased the use of resin-based composite materials for both anterior and posterior restorations [1]. Their favorable optical characteristics and mechanical properties enable close reproduction of the appearance of natural dentition. Traditionally, achieving optimal esthetics has required the use of multiple composite shades and varying opacities applied in incremental layers. Although effective, this technique is technique-sensitive, time-consuming, and often associated with increased clinical complexity and treatment costs [2,3].

To make the shade selection step easier, there has been an introduction of newer single-shade composite materials. Single-shaded composite materials match the color of the tooth to be restored, exhibiting a phenomenon called “chameleon effect,” which is the ability of a material to combine and obtain a color similar to its surrounding structure [4].

Omnichroma (Tokuyama Dental, Tokyo, Japan) is a novel single-shaded composite with uniformly sized supra-nano spherical fillers designed for use in most restorative clinical cases with high color matching ability [5]. Vittra APS Unique (FGM Dental Group, Joinville, Santa Catarina, Brazil) is also a single-shaded composite having a blending effect with surrounding tooth structure, high polymerization strength, which allows for a higher conversion rate and better mechanical properties [6].

Long-term esthetic success of composite restorations depends largely on their ability to resist discoloration. Color changes may occur intrinsically because of alterations within the resin matrix or extrinsically through exposure to pigments from beverages, foods, and other environmental factors. Oral hygiene practices, dietary habits, and smoking all influence the degree of extrinsic staining [7]. Therefore, maintaining color stability is essential for the longevity and patient acceptance of tooth-colored restorations

There are fewer studies comparing the effect of different beverages on the color stability of different single-shaded composite materials. Hence, the aim of this study is to compare the effect of beverages on the color stability of two single-shaded composite resin materials. The null hypothesis tested was: (1) there would be no significant difference in color stability between the two single-shade composite resin materials, and (2) immersion in different dietary beverages would not significantly alter their color.

## Materials and methods

This in vitro study was conducted to comparatively evaluate the effect of various commonly consumed beverages on the color stability of two single-shade composite resin materials.

Specimen preparation

**Figure 1:**
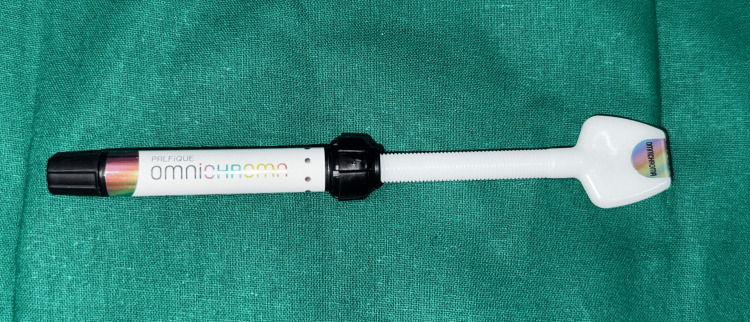
Omnichroma composite Omnichroma (Tokuyama Dental, Tokyo, Japan)

**Figure 2:**
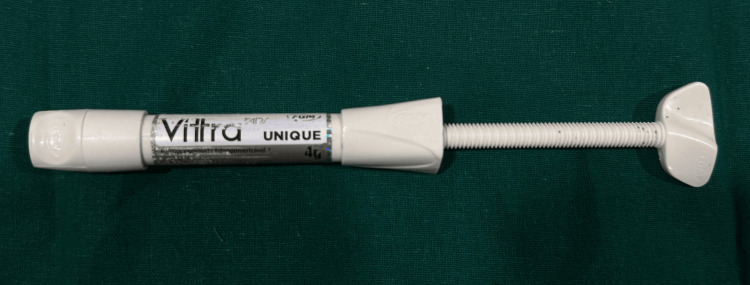
Vittra APS Unique composite material Vittra APS Unique (FGM Dental Group, Joinville, Santa Catarina, Brazil)

A total of 96 disc-shaped composite specimens were prepared for this study. The specimens were fabricated from two commercially available single-shade resin composites: Omnichroma (n = 48) (Figure 1), Vittra APS Unique (n = 48) (Figure 2).

The composition of these single-shade composite resins is presented in Table 1.

**Table 1:** Name, manufacturer and composition of single-shade composite materials used in the present study

Sl no.	Material	Composition
1	Omnichroma composite resin (Tokuyama Dental, Tokyo, Japão)	Supra-nanospherical fillers of uniform size (260 nm spherical SiO2-ZrO2). Contains 79% by weight (68% by volume) of spherical zirconium silica filler (average particle size: 0.3 μm, range 0.2 to 0.4 μm) and composite material. Contains 1,6-bis (methacrylo-ethyloxycarbonylamino) trimethyl hexane (UDMA), triethylene glycol-dimethacrylate (TEGDMA), mequinol, dibutylhydroxytoluene and UV absorber.
2	Vittra APS Unique composite resin (FGM Dental Group, Joinville, Santa Catarina, Brazil)	Total nano-hybrid content of inorganic filler of 72–80% by weight (52–60% by volume). It does not contain Bis-GMA or Bis-EMA in its formulation, following the current trend towards bisphenol A-free products (BPA). Its basic composition contains the active ingredients: mixture of methacrylate monomers, photo initiator composition (APS), co-initiators, stabilizers and silane; and inactive ingredients: boron-aluminum-silicate glass.

**Figure 3:**
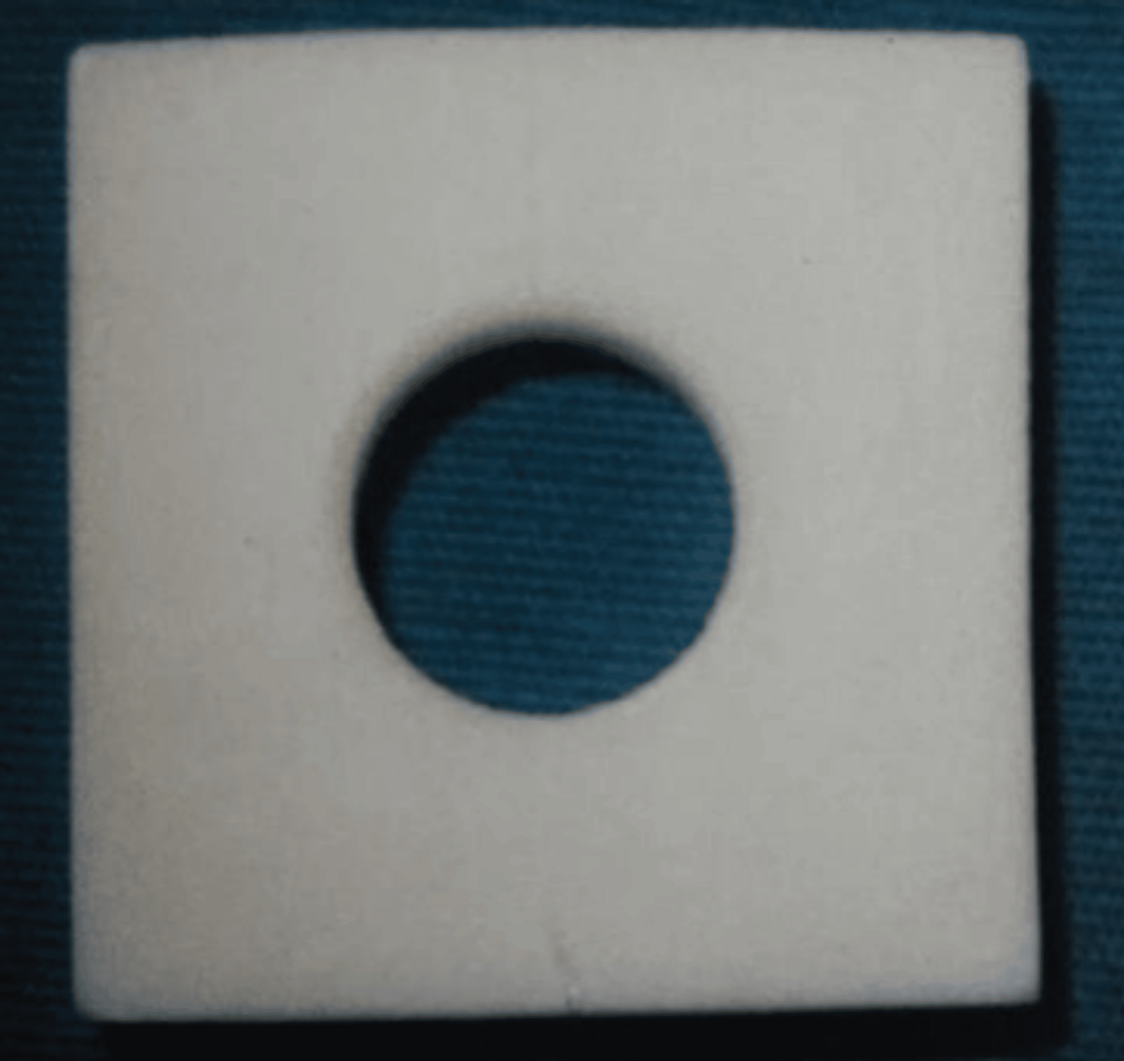
Polytetrafluoroethylene mold (Teflon) used for used for fabrication of resin composite discs Teflon (The Chemours Company, Wilmington, DE, USA)

To ensure standardized dimensions, a customized polytetrafluoroethylene (PTFE) mold was utilized to fabricate discs with a diameter of 10 mm and a thickness of 2 mm (Figure 3). The mold was custom-fabricated by precision-machining a solid PTFE block using a lathe to create uniform, cylindrical wells with an exact internal diameter of 10 mm and a depth of 2 mm. The composite resin materials were carefully packed into the preformed mold. To extrude excess material, prevent the formation of an oxygen-inhibited layer, and achieve a highly flat and smooth surface, a transparent polyester matrix strip and a rigid glass slab were placed over the mold and pressed with light digital pressure. Incremental layers were condensed into a mold. Each increment was cured for 40 seconds using a light-emitting diode (LED) curing unit (Ivoclar Vivadent, Glattpark, Switzerland) emitting a standard irradiance of 1200 mW/cm². Following polymerization, all specimens were finished and polished uniformly to simulate a clinically polished restoration, ensuring that surface roughness would not be an uncontrolled variable in color absorption.

Preparation of staining solutions and immersion protocol

**Figure 4:**
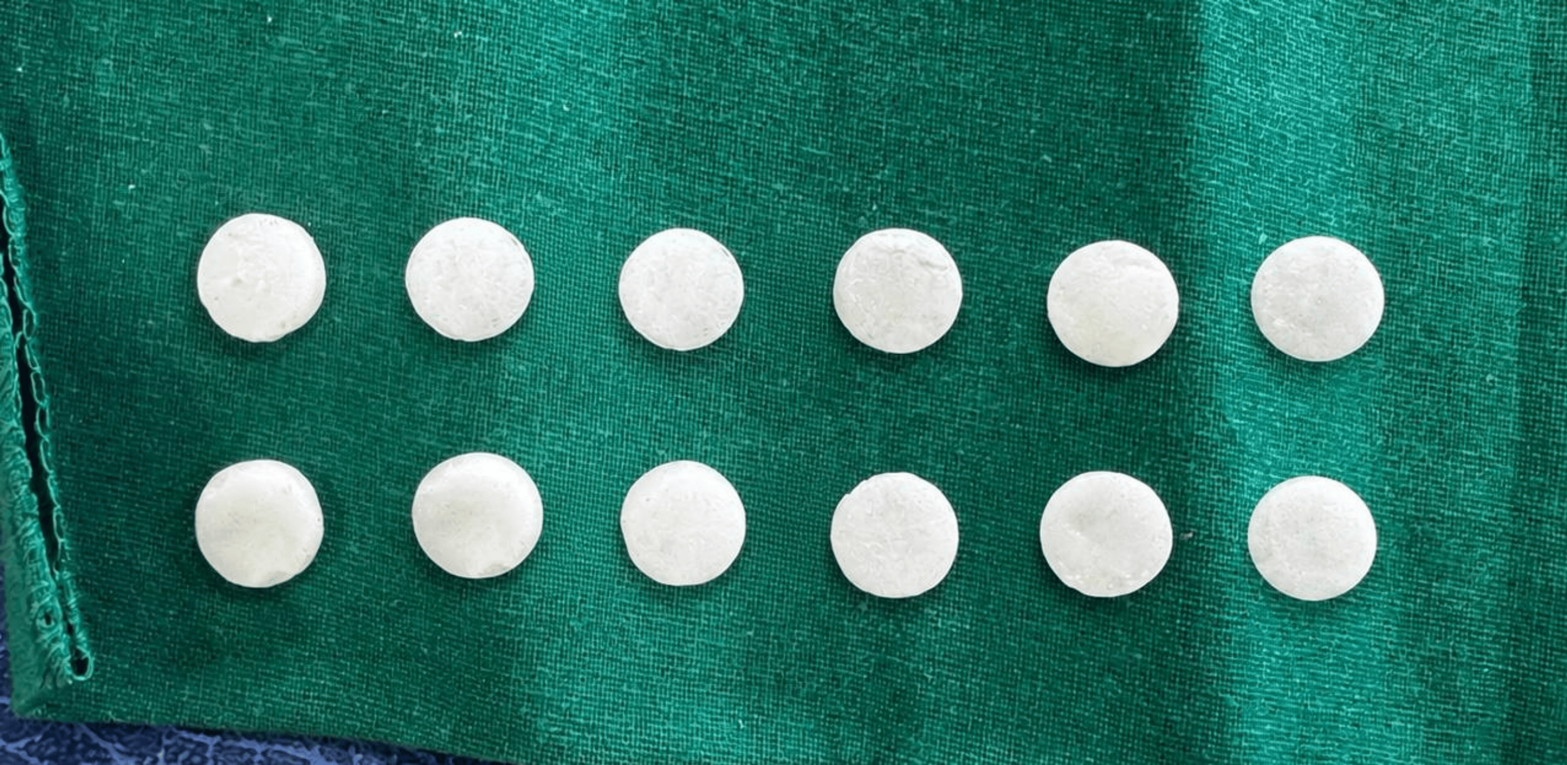
Representative image of the prepared disc-shaped Omnichroma composite specimens (10 mm × 2 mm) at baseline, prior to the immersion protocol Omnichroma (Tokuyama Dental, Tokyo, Japan)

**Figure 5:**
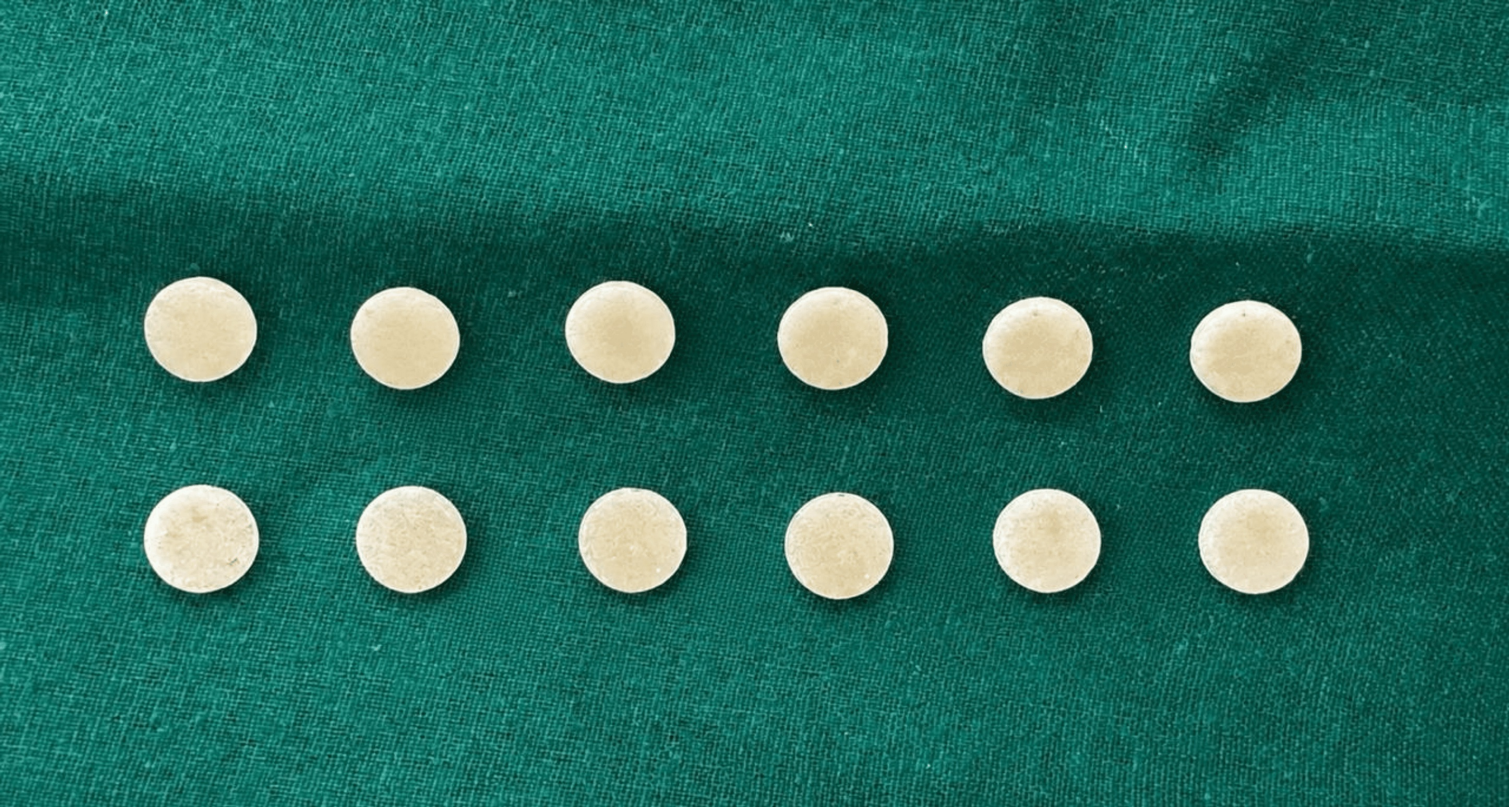
Representative image of the prepared disc-shaped Vittra APS Unique composite specimens (10 mm × 2 mm) at baseline, prior to the immersion protocol Vittra APS Unique (FGM Dental Group, Joinville, Santa Catarina, Brazil)

To simulate the exposure to dietary colorants in the oral cavity, the 48 specimens of each composite brand were randomly subdivided into four experimental groups (n = 12 per subgroup) (Figures 4, 5) based on the immersion medium: Group A (Control): Artificial saliva, Group B: Turmeric powder water solution(prepared by dissolving 1 g of turmeric powder in 1000 mL of boiling distilled water, stirred for 10 minutes, and filtered), Group C: Tea (Brooke Bond Red Label Natural Care Tea, Hindustan Unilever Limited, Mumbai, Maharashtra, India) (prepared by dissolving 10 g of Brooke Bond Red Label Natural Care Tea in 300 mL of boiling distilled water, brewed for 10 minutes, and filtered) and Group D: Soft Drink (Coca-Cola, The Coca-Cola Company, Atlanta, GA, USA). The specimens from both composite groups were continuously immersed in their respective beverages for a period of 28 days. While continuous immersion does not perfectly replicate the dynamic flushing effect of saliva in the oral cavity, it serves as a standard accelerated in vitro aging model. Based on previous literature, 24 hours of continuous in vitro staining correlates to approximately one month in vivo. Thus, this 28-day continuous immersion protocol was specifically selected to simulate the extreme scenario of approximately two years and six months of clinical aging.

Spectrophotometric color evaluation

Color stability was quantitatively assessed using a portable spectrophotometer (FRU Model WR-10, Shenzhen, China). Prior to each measurement session, the device was calibrated according to the manufacturer’s recommendations. Before any readings were taken, the specimens were gently rinsed with distilled water and blot dried. To ensure strict reproducibility and eliminate background light interference, all measurements were performed with the discs placed on a standardized white background. To record the color coordinates, the flat measuring base of the spectrophotometer, which features an 8 mm measuring aperture, was placed flush against the prepared composite discs (10 mm diameter). Because the 8 mm aperture evaluated the vast majority of the specimen's surface area simultaneously, a single, precise reading was recorded for each individual sample. Baseline color measurements were recorded for each specimen prior to any immersion. Following the 28-day immersion period, the specimens were measured again to determine the degree of color change. Color parameters were recorded using the Commission Internationale de l'Eclairage (CIELAB) color system, which maps color across three spatial coordinates: L* (lightness), a* (red-green axis), and b* (yellow-blue axis). The overall color difference for each specimen, representing the degree of color change from baseline to post-immersion, was computed employing the standard formula:

$\Delta E_{ab}^{*} = \sqrt{(\Delta L^{*})^{2} + (\Delta a^{*})^{2} + (\Delta b^{*})^{2}}$

where ΔE = overall color difference, ΔL* = change in lightness, Δa* = change along the red-green axis, and Δb* = change along the yellow-blue axis.

Statistical analysis

All collected data were systematically compiled in Microsoft Excel (Microsoft Corporation, Redmond, WA, USA) and subjected to statistical analysis using IBM SPSS Statistics, version 21 (IBM Corp., Armonk, NY, USA). The normality of the data distribution was first evaluated using the Shapiro-Wilk test. Because the data did not follow a normal distribution, non-parametric statistical tests were applied. Intragroup comparisons - to assess the significance of color change between the baseline and post-immersion values-were conducted using the Wilcoxon signed-rank test. Intergroup comparisons - to evaluate the differences between the Omnichroma and Vittra APS Unique composites - were performed using the Mann-Whitney U test. For all statistical evaluations, a p-value of 0.05 was considered statistically significant.

## Results

The color stability of the two single-shade composite resins, Omnichroma (Group 1) and Vittra APS Unique (Group 2), was quantitatively assessed by measuring the CIELAB parameters - L* (lightness), a* (red-green axis), and b* (yellow-blue axis)- and calculating the overall color difference (ΔE) following a 28-day immersion in artificial saliva, turmeric solution, tea, and soft drinks.

Artificial saliva

Both composites showed significant intragroup changes in L*, a*, and b* values after immersion. Lightness (L*) increased in both materials, indicating a lighter appearance. The shift in a* toward negative values indicated a move toward greenish tones, while reduced b* values indicated decreased yellowness. Overall color change (ΔE) was significantly higher in Omnichroma (17.37±2.79) compared to Vittra APS (10.58±0.11), showing greater discoloration in Omnichroma (Table 2).

**Table 2:** Comparison of CIELAB color parameters (L*, a*, b*) and overall color change (ΔE) of Omnichroma and Vittra APS Unique composites at baseline and after immersion in artificial saliva † indicates that p-value is significant when p-value is less than 0.05 CIELAB = Commission Internationale de l'Eclairage Omnichroma (Tokuyama Dental, Tokyo, Japan); Vittra APS Unique (FGM Dental Group, Joinville, Santa Catarina, Brazil)

Variables	Omnichroma (Mean ± SD)	Vittra APS Unique (Mean ± SD)	p-value
Artificial Saliva L Baseline	62.98 ± 4.60	64.95 ± 0.12	1.000
Artificial Saliva L After	74.84 ± 0.71	75.51 ± 0.22	0.032 †
p-value	0.002 †	0.002 †	-
Artificial Saliva a Baseline	2.35 ± 0.07	-.83 ± 0.14	<0.001 †
Artificial Saliva a After	-.28 ± 0.71	-.90 ± 0.49	0.032 †
p-value	0.002 †	0.147	-
Artificial Saliva b Baseline	16.38 ± 0.88	3.84 ± 0.72	<0.001 †
Artificial Saliva b After	4.25 ± 0.51	3.95 ± 0.04	1.000
p-value	0.002 †	0.147	-
ΔE	17.37 ± 2.79	10.58 ± 0.11	<0.001 †

Turmeric

Turmeric produced the most pronounced color alterations. Significant intragroup changes were observed in all CIELAB parameters for both materials. Increased a* and b* values indicated strong red and yellow staining. ΔE was significantly greater in Vittra APS (30.00±4.69) than Omnichroma (18.60±2.47), demonstrating higher susceptibility of Vittra APS to turmeric staining (Table 3). Turmeric showed the highest staining potential among all beverages.

**Table 3:** Comparison of CIELAB color parameters (L*, a*, b*) and overall color change (ΔE) of Omnichroma and Vittra APS Unique composites at baseline and after immersion in turmeric † indicates that p-value is significant when p-value is less than 0.05 CIELAB = Commission Internationale de l'Eclairage Omnichroma (Tokuyama Dental, Tokyo, Japan); Vittra APS Unique (FGM Dental Group, Joinville, Santa Catarina, Brazil)

Variables	Omnichroma (Mean ± SD)	Vittra APS Unique (Mean ± SD)	p-value
Turmeric L Baseline	60.46 ± 2.09	63.91 ± 1.37	<0.001 †
Turmeric L After	71.36 ± 0.48	63.52 ± 0.31	<0.001 †
p-value	0.007 †	0.147	-
Turmeric a Baseline	2.67 ± 0.03	-.56 ± 0.13	<0.001 †
Turmeric a After	9.31 ± 2.14	14.25 ± 0.49	<0.001 †
p-value	0.002 †	0.002 †	-
Turmeric b Baseline	18.90 ± 0.02	4.31 ± 0.59	<0.001 †
Turmeric b After	31.46 ± 4.77	30.18 ± 5.03	0.032 †
p-value	0.002 †	0.002 †	-
ΔE	18.60 ± 2.47	30.00 ± 4.69	<0.001 †

Tea

Tea caused significant darkening in both composites, reflected by decreased L* values. Increases in a* and b* values indicated a shift toward red and yellow hues. Vittra APS exhibited higher ΔE (17.80±4.38) compared to Omnichroma (5.98±1.39), indicating greater discoloration, although both materials showed significant intragroup color change (Table 4).

**Table 4:** Comparison of CIELAB color parameters (L*, a*, b*) and overall color change (ΔE) of Omnichroma and Vittra APS Unique composites at baseline and after immersion in tea † indicates that p-value is significant when p-value is less than 0.05 CIELAB = Commission Internationale de l'Eclairage Omnichroma (Tokuyama Dental, Tokyo, Japan); Vittra APS Unique (FGM Dental Group, Joinville, Santa Catarina, Brazil)

Variables	Omnichroma (Mean ± SD)	Vittra APS Unique (Mean ± SD)	p-value
Tea L Baseline	63.30 ± 0.83	63.91 ± 1.37	<0.001 †
Tea L After	58.94 ± 1.49	55.72 ± 1.68	<0.001 †
p-value	0.002 †	0.002 †	-
Tea a Baseline	2.39 ± 0.00	-0.56 ± 0.130	<0.001 †
Tea a After	3.66 ± 0.03	6.90 ± 1.59	<0.001 †
p-value	0.002 †	0.002 †	-
Tea b Baseline	16.38 ± 0.54	3.85 ± 0.37	<0.001 †
Tea b After	12.95 ± 0.00	17.51 ± 3.22	<0.001 †
p-value	0.002 †	0.002 †	-
ΔE	5.98± 1.39	17.80 ± 4.38	<0.001 †

Soft drinks

Both materials demonstrated significant intragroup changes in L*, a*, and b* values. However, the overall color change was comparatively smaller than that of the other beverages. ΔE values were similar between Omnichroma (7.79±3.49) and Vittra APS (7.22±1.41), indicating comparable staining effects and no significant intergroup difference (Table 5).

**Table 5:** Comparison of CIELAB color parameters (L*, a*, b*) and overall color change (ΔE) of Omnichroma and Vittra APS Unique composites at baseline and after immersion in soft drinks † indicates that p-value is significant when p-value is less than 0.05 CIELAB = Commission Internationale de l'Eclairage Omnichroma (Tokuyama Dental, Tokyo, Japan); Vittra APS Unique (FGM Dental Group, Joinville, Santa Catarina, Brazil)

Variables	Omnichroma (Mean ± SD)	Vittra APS Unique (Mean ± SD)	p-value
Soft Drinks L Baseline	60.61 ± 0.00	65.09 ± 1.75	<0.001 †
Soft Drinks L After	65.25 ± 1.28	70.95 ± 0.19	<0.001 †
p-value	0.002 †	0.002 †	-
Soft Drinks a Baseline	2.63 ± 0.09	-.86± 0.10	<0.001 †
Soft Drinks a After	1.70 ± 0.88	.51± 0.01	<0.001 †
p-value	0.147	0.002 †	-
Soft Drinks b Baseline	18.37 ± 0.50	4.05 ± 0.43	<0.001 †
Soft Drinks b After	12.30 ± 2.84	7.82 ± 0.06	<0.001 †
P-value	0.002 †	0.002 †	-
ΔE	7.79 ± 3.49	7.22 ± 1.41	1.000

Effect of beverages on Omnichroma

When evaluating the Omnichroma composite individually, the material demonstrated varying susceptibilities to the immersion media. The highest overall color change was observed after immersion in the turmeric solution (ΔE = 18.60 ± 2.47). Notably, when comparing the effect of artificial saliva against the dietary beverages, continuous immersion in the non-staining artificial saliva caused a surprisingly high intrinsic color shift (ΔE = 17.37 ± 2.79) that was second only to turmeric, primarily driven by a significant increase in lightness (L*). Conversely, the acidic environment of soft drinks (ΔE = 7.79 ± 3.49) and the dark pigments of tea (ΔE = 5.98 ± 1.39) produced significantly less severe color alterations in the Omnichroma specimens.

The unexpectedly higher ΔE observed in the Omnichroma artificial saliva group compared to tea immersion appears to be predominantly related to intrinsic optical alterations rather than extrinsic staining. A substantial increase in L* values indicated lightening of the specimens during prolonged water storage. Because Omnichroma depends on structural color technology and high translucency for its chameleon effect, water sorption may alter the refractive index relationship between the resin matrix and supra-nano spherical fillers, producing marked optical shifts even in the absence of pigments. Therefore, the elevated ΔE in artificial saliva should be interpreted as hydration-induced intrinsic color instability rather than true staining.

Effect of beverages on Vittra APS Unique

When evaluating the Vittra APS Unique composite individually, a distinctly different staining hierarchy was observed. Turmeric produced the most profound and severe discoloration (ΔE = 30.00 ± 4.69), followed by significant darkening and staining caused by tea (ΔE = 17.80 ± 4.38). In comparison to these potent dietary beverages, immersion in artificial saliva resulted in a milder, intrinsic color change (ΔE = 10.58 ± 0.11). Finally, soft drinks caused the least amount of color degradation for this specific composite (ΔE = 7.22 ± 1.41).

Overall staining potential among the beverages evaluated, the materials exhibited varying degrees of susceptibility to discoloration. The overarching trend for staining potential across both single-shade composite resins was Turmeric >> Tea >> Soft drinks, with soft drinks producing the least amount of severe discoloration. However, significant intrinsic color shifts were also observed in the artificial saliva (control) group. Even without the presence of a dietary staining agent, continuous immersion in artificial saliva caused both composites to undergo significant intragroup changes; specifically, an increase in lightness (L*), a shift toward greenish tones (negative a*), and decreased yellowness (reduced b*). In this non-staining control medium, Omnichroma demonstrated a significantly higher overall color change (ΔE=17.37±2.79) compared to Vittra APS Unique (ΔE=10.58±0.11), indicating a substantial inherent discoloration likely driven by water sorption over the 28-day period.

## Discussion

The long-term clinical success of direct composite restorations depends not only on mechanical durability but also on sustained esthetic performance. Single-shade composite resins were developed to simplify shade selection while maintaining excellent esthetic integration with surrounding tooth structure. Their ability to adapt visually is based on structural color principles and controlled translucency, which together produce the chameleon effect [1,3,8]. However, a primary concern for the clinical longevity of any aesthetic restoration is its resistance to discoloration in the oral environment. The present in vitro study evaluated the color stability of two single-shade composites, Omnichroma and Vittra APS Unique, following immersion in artificial saliva, turmeric solution, tea, and soft drinks. Because significant color changes were observed across all experimental groups, the null hypothesis that different beverages do not affect the color stability of the tested materials was rejected.

To simulate prolonged intraoral exposure, a 28-day continuous immersion protocol was utilized in this study. This duration has been established in the dental literature as clinically equivalent to approximately two years and six months of in vivo clinical aging [9]. Over this simulated aging period, turmeric produced the most severe and pronounced color alterations among all tested media, followed closely by tea. This is consistent with a previous study by Checchi et al., who demonstrated that turmeric-based solutions and staining food substances cause the most significant pigmentation and color instability in single- and multi-shade composite resins [7]. The severe discoloration caused by turmeric is largely attributed to the compatibility of its highly polar yellow pigments and polyphenolic structures with the organic matrix of the composite resins, allowing the stains to absorb deeply into the material.

Between the two single-shade materials, Vittra APS Unique exhibited a significantly higher overall color change (ΔE) and greater susceptibility to staining from both turmeric and tea compared to Omnichroma. The superior color stability of Omnichroma against heavy dietary stains like tea strongly aligns with the findings of a study conducted by Kalander et al. and Rohym et al., who both reported that Omnichroma demonstrates higher color stability and surface resistance against common beverages like coffee and tea compared to other contemporary composite resins [10,11]. This resistance may be attributed to Omnichroma’s specific "smart chromatic technology," which utilizes uniformly sized 260 nm supra-nano spherical silica-zirconia fillers and a highly cross-linked resin matrix [5]. The uniformity of these fillers provides a denser barrier against the penetration of polar stains compared to the nanohybrid structure of Vittra APS Unique. Furthermore, studies evaluating the color matching of single-shade composites with different background shades suggest that filler composition heavily dictates how effectively these materials maintain color stability over time [12].

Conversely, soft drinks induced the least severe discoloration among the dietary beverages, with no significant intergroup difference between Omnichroma and Vittra APS Unique. The primary mechanism of composite degradation from aerated soft drinks is typically driven by their low pH and acidic nature, which causes hydrolytic degradation, surface erosion, and microcracks at the filler-matrix interphase rather than heavy pigment deposition [13]. Because both materials experienced similar matrix degradation under these acidic conditions without the heavy absorption of dark colorants, their resulting ΔE values were comparatively low and statistically similar.

Interestingly, in the artificial saliva (control) group, Omnichroma demonstrated a significantly higher ΔE than Vittra APS Unique, indicating a slight intrinsic color shift (becoming lighter) even without a staining agent. Because artificial saliva does not contain pigments, this color change is likely due to the water sorption characteristics of the resin matrix over the 28-day wet storage. As water penetrates the interstitial spaces, it causes swelling and plasticization. Rosa et al. highlighted that color matching and color recovery in large single-shade composite restorations are highly dependent on substrate interaction, and intrinsic shifts can occur as the material absorbs water and its refractive index changes [14]. Tepe et al. similarly noted in spectrophotometric evaluations that the high translucency required for single-shade composites to mimic natural tooth color can also render them vulnerable to reflecting severe intrinsic shifts over time [15].

Interpretation of color change values should consider clinical perceptibility and acceptability thresholds. Previous studies have reported that ΔE values greater than approximately 1.2 may be perceptible to trained observers, while values exceeding 2.7-3.3 are generally considered clinically unacceptable. In the present study, nearly all immersion groups demonstrated ΔE values above commonly accepted clinical acceptability thresholds, particularly turmeric and tea groups, indicating that prolonged exposure to these beverages may produce clinically visible discoloration. Therefore, although both restorative materials demonstrated varying degrees of discoloration resistance, several immersion conditions produced color changes exceeding clinically acceptable limits.

The limitations of this in vitro study include the continuous immersion of the specimens, which represents an extreme clinical scenario. In a dynamic oral environment, restorations are continuously flushed by saliva, which dilutes staining factors and buffers acidic pH levels. A notable limitation of the present study is its exclusive focus on optical color stability without the concurrent evaluation of other physical and mechanical parameters. The literature indicates that variables such as surface roughness, microhardness, and water sorption are deeply interconnected with a composite resin's susceptibility to extrinsic staining. While isolating color stability allowed for a focused spectrophotometric assessment of the single-shade 'chameleon effect,' the lack of correlated surface topography analysis means the exact mechanism of degradation (e.g., matrix erosion versus simple pigment adsorption) cannot be fully authenticated. Furthermore, the present study did not incorporate thermocycling or simulated tooth-brushing, which are known to induce microcracks and surface wear that heavily influence stain retention. The use of flat, disc-shaped specimens also fails to replicate the complex convex and concave anatomy of clinical restorations, where stains typically accumulate. Moreover, while this study assessed the impact of common dietary beverages, it did not evaluate the severe discoloration potential of patient habits such as tobacco chewing and smoking. Future in vivo clinical trials incorporating mechanical wear, thermocycling, dynamic salivary flow, and the concurrent evaluation of surface roughness and microhardness are necessary to validate these findings. Nonetheless, clinicians must consider patient dietary habits such as heavy consumption of turmeric or tea when selecting single-shade composites, as these materials exhibit varying degrees of long-term color stability.

## Conclusions

It is concluded that single-shade composite resins possess excellent initial shade-matching properties; their long-term color stability is significantly influenced by dietary beverages. The severity of the discoloration was highly dependent on the type of immersion medium, with the staining potential ranking as follows: Turmeric > Tea > Soft drinks.

Between the two evaluated materials, Omnichroma demonstrated superior color stability and significantly higher resistance to severe dietary colorants (turmeric and tea) compared to Vittra APS Unique. Conversely, soft drinks produced the least discoloration among the beverages, showing comparable, milder degradation effects on both composites. Furthermore, even in a non-staining control medium (artificial saliva), both materials underwent intrinsic color shifts, highlighting the impact of water sorption and hydrolytic degradation over time. To ensure long-term aesthetic success, clinicians must consider the patient's dietary habits, especially the frequent intake of turmeric and tea, when selecting restorative materials, and advise patients on the potential for staining.
